# Expression, Localization, and Effect of High Salt Intake on Electroneutral Na^+^/HCO_3_^–^ Cotransporter NBCn2 in Rat Small Intestine: Implication in Intestinal NaCl Absorption

**DOI:** 10.3389/fphys.2019.01334

**Published:** 2019-10-29

**Authors:** Jin-Lin Wang, Lei Zhao, Jun Zhu, Deng-Ke Wang, Mei-Juan Ren, Meng Wang, Ying Liu, Walter F. Boron, Li-Ming Chen

**Affiliations:** ^1^Key Laboratory of Molecular Biophysics of the Ministry of Education, School of Life Science and Technology, Huazhong University of Science and Technology, Wuhan, China; ^2^Department of Obstetrics, Maternal and Child Health Hospital of Hubei Province, Wuhan, China; ^3^Department of Physiology and Biophysics, Case Western Reserve University School of Medicine, Cleveland, OH, United States

**Keywords:** alternative splicing, bicarbonate transporter, epithelium, NaCl absorption, small intestine

## Abstract

The electroneutral Na^+^/HCO_3_^–^ cotransporter NBCn2 (SLC4A10) of solute carrier family 4 (SLC4) plays important physiological and pathological roles in the body. Our previous study showed that NBCn2 is expressed on the protein level in the small intestine of rat. Here, by reverse-transcription polymerase chain reaction (PCR), we identified a novel full-length NBCn2 variant, i.e., NBCn2-K, from rat small intestine. By pH_i_ measurement with *Xenopus* oocytes, the activity of NBCn2-K is not significantly different from NBCn2-G. By western blotting, NBCn2 and the Na^+^/H^+^ exchanger NHE3 (SLC9A3) are predominantly expressed in the jejunum of rat small intestine. By immunofluorescence, NBCn2 and NHE3 are localized at the apical domain of the jejunum. NaCl overload decreases the expression of NBCn2 by 56% and that of NHE3 by 40% in the small intestine. We propose that NBCn2 is involved in the transepithelial NaCl absorption in the small intestine, and that the down-regulation of NBCn2 by NaCl represents an adaptive response to high salt intake in rat.

## Introduction

Na^+^/HCO_3_^–^ cotransporter NBCn2, the product of *SLC4A10* gene, plays significant physiological and pathological roles in the body. In human, genetic abnormality in locus 2q24 spanning *SLC4A10* is associated with complex epilepsy, mental retardation, autism spectra, cognitive disabilities, and hearing impairment (Sebat et al., 2007; Gurnett et al., 2008; Krepischi et al., 2010; Belengeanu et al., 2014; Nilsson et al., 2017; Zhao et al., 2018). In mouse, genetic disruption of *Slc4a10* reduces neuronal excitability, resulting in increased seizure threshold (Jacobs et al., 2008), impairs the visual acuity and contrast sensitivity (Hilgen et al., 2012), and causes hearing loss (Potter et al., 2016; Huebner et al., 2019). A Cohort study shows that the expression of *SLC4A10* is associated with the age-dependent increase in blood plasma interleukin IL6, an indicator of inflammation (Pilling et al., 2015). Finally, a GWAS meta-analysis shows that *SLC4A10* is involved in the regulation of plasma osmolarity in human (Boger et al., 2017).

NBCn2 (*aka* NCBE) was originally characterized as a Na^+^-driven Cl^–^-HCO_3_^–^ exchanger (Wang et al., 2000). It is clear that NBCn2 mediates the electroneutral cotransport of Na^+^ and HCO_3_^–^. However, it remains controversial whether the cotransport of Na^+^ and HCO_3_^–^ mediated by NBCn2 is associated with an efflux of intracellular Cl^–^. By surface Cl^–^ measurement with *Xenopus* oocytes, NBCn2, like NBCn1, causes no increase in the surface concentration of Cl^–^ ([Cl^–^]_s_) upon the introduction of CO_2_/HCO_3_^–^ (Parker et al., 2008). This observation is in striking contrast to the significant rise in [Cl^–^]_s_ in cells expressing AE1 (SLC4A1) or NDCBE (SLC4A8), both of which are established Cl^–^/HCO_3_^–^ exchangers either Na^+^ independent (AE1) or Na^+^ dependent (NDCBE). The lack of change in [Cl^–^]_s_ argues against the idea that NBCn2 is an Na^+^-driven Cl^–^/HCO_3_^–^ exchanger. It is intriguing that depletion of intracellular Cl^–^ eliminates the Na^+^-dependent pH_i_ recovery in 3T3 cells expressing NBCn2 (Damkier et al., 2010). One likely explanation is that intracellular Cl^–^ ion represents a regulatory factor that is essential for the function of NBCn2.

**FIGURE 1:**
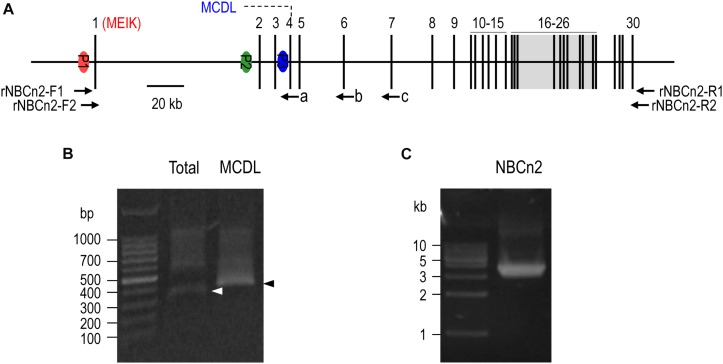
5′-RACE and RT-PCR analysis of *Slc4a10* expression in small intestine of rat. **(A)** Diagram of structure of rat *Slc4a10* gene. Rat *Slc4a10* contains 30 exons and three promoters: promoter P1 (upstream of exon 1), P2 (located in the intron between exons 1 and 2), and P3 (located in the intron between exons 3 and 4). Exon 1 contains the coding sequence for unique Nt of MEIK-NBCn2. Exon 4 contains the coding sequence for the unique Nt of MCDL-NBCn2. Exons 6-26 boxed in gray encode the transmembrane domain of NBCn2. **(B)** Agrose gel analysis of 5′-RACE of *Slc4a10* from small intestine. **(C)** Agrose gel analysis of RT-PCR product of full-length *Slc4a10* transcripts from small intestine. Arrows a, b, and c in panel **(A)** indicate the approximate positions of the primers used for 5′-RACE. Arrowheads in panel **(B)** indicate the target bands of PCR products of *Slc4a10*.

The mammalian *SLC4A10* gene contains multiple promoters (Figure 1A), controlling the expression of two groups of full-length NBCn2 variants differing in the extreme amino-terminal (Nt) end. The first group of NBCn2 variants starts with “MEIK” (the initial four residues), expressed under the control of the distal promoter P1 of *SLC4A10*. MEIK-NBCn2 is most abundantly expressed in the central nervous system (CNS) (Wang et al., 2000; Parker et al., 2008). In the CNS, NBCn2 is abundant in neurons throughout the brain and in the retina (Chen et al., 2008; Jacobs et al., 2008; Hilgen et al., 2012) as well as in the epithelium of the choroid plexus (Chen et al., 2008; Jacobs et al., 2008; Liu et al., 2010). In the brain, NBCn2 plays an important role in the modulation of neuronal excitability (Jacobs et al., 2008) and short term plasticity (Sinning et al., 2015). MEIK-NBCn2 is also involved in the secretion of cerebro spino fluid (Jacobs et al., 2008). Elsewhere, MEIK-NBCn2 is expressed, at relatively lower levels, in the kidney and reproductive tract tissues (Liu et al., 2013; Guo et al., 2017). In the kidney, MEIK-NBCn2 is expressed at the basolateral membrane of the medullary thick ascending limb and inner medullary collecting duct (Guo et al., 2017).

The second group of NBCn2 variants is derived from the proximal promoters (P2 or P3) of *SLC4A10*. The initial Nt end of this group of NBCn2 contains a minor species-specific variation due to genomic variations. In rat, this group of NBCn2 starts with “MCDL”. The human homologs start with “MQSL”, whereas the mouse homologs start with “MQPG” (Liu et al., 2013; Wang et al., 2015). In rat, MCDL-NBCn2 is predominantly expressed at the apical membrane of the proximal tubules in the kidney (Liu et al., 2013; Guo et al., 2017). By mediating direct HCO_3_^–^ uptake, this MCDL-NBCn2 contributes to HCO_3_^–^ reabsorption, a strategy distinct from the conventional one depending on proton secretion via apical Na^+^-H^+^ exchanger NHE3 and proton pump (Guo et al., 2017).

Finally, in rat, *Slc4a10* is able to produce a group of transcripts predicted to express NBCn2 variants that are N-terminally truncated by about one fourth of the conserved regions of the cytosolic Nt domain (Wang et al., 2015). These Nt-truncated NBCn2 variants, if expressed, would be inactive in terms of Na^+^/HCO_3_^–^ cotransport.

In the previous study (Liu et al., 2013), NBCn2 protein is identified in the small intestine of rat by western blotting using an antibody against the unique Nt of NBCn2 starting with MCDL. In the present study, we cloned from rat small intestine a full-length cDNA that is transcribed under the control of distal promoter of *Slc4a10*. This cDNA is predicted to express an NBCn2 variant containing both “MEIK” and “MCDL”. Immunofluorescence study shows that NBCn2 is expressed at the apical domain of the small intestinal epithelia. The abundance of NBCn2 in small intestine is down-regulated by high salt intake. We propose that NBCn2 is involved in NaCl absorption in the small intestine.

## Materials and Methods

### Antibodies

Rabbit polyclonal anti-MCDL and anti-MEIK have been described and characterized previously (Liu et al., 2013; Guo et al., 2017). Rabbit polyclonal anti-NBCn1 was custom-made against an immunogen “ENAKVTRPNMSPEKPVSVTC” by Genscript (Nanjing, CN). Rabbit anti-NHE3 (cat#ab-95299) and mouse anti-α1 (cat#ab-7671) against the α1 subunit of Na^+^-K^+^ pump (*aka* Na^+^-K^+^-ATPase) were purchased from Abcam (HK, China). Mouse anti-actin was purchased from Beyotime (Cat#AA128, Haimen, Jiangsu, China). HRP-conjugated secondary antibodies were from Beyotime. Alexa Fluor^®^ 488 AffiniPure goat anti-mouse (cat# 115-545-003) was from Jackson ImmunoResearch (West Grove, PA, United States). Dylight 549 goat anti-rabbit (cat#E032320-01) was from EarthOx (Millbrae, CA, United States).

### Animals

Adult Sprague Dawley (SD) rats (weighted ∼200 g) were purchased from the Hubei Provincial Center for Disease Control (Wuhan, China). The procedures for the experiments with animals were approved by the Institutional Committee on Animal Care and Use at Huazhong University of Science and Technology (Animal Study Proposal #2016114). The rats were randomly assigned into two groups. The high salt group was supplied with water containing 1.5% (w/v) NaCl. The control was fed with plain tap water. The rats had free access to rodent chow. The rats were treated for 7 days, then anesthetized by subcutaneous injection of pentobarbital sodium and sacrificed for tissue collection. The tissue was frozen in liquid nitrogen immediately and stored at −80°C until used.

### 5′-RACE and Full-Length cDNA Cloning

Total RNA was isolated from rat small intestine with TRIzol^®^ reagent (Life Technologies Corporation, Carlsbad, CA, United States) following the manufacturer’s instructions. 5′-rapid amplification of cDNA ends (5′-RACE) was performed with Clontech SMARTer^®^ RACE 5′/3′ Kit according to manufacturer’s instructions. The first-strand cDNA was synthesized with 5′ RACE CDS primers and SMARTer II A Oligonucleotide provided by the kit. The 5′-UTR of *Slc4a10* transcripts was amplified by nested polymerase chain reaction (PCR). The first round PCR was performed with the sense Universal Primer of the kit and the antisense primer “c” 5′-GTGCTCCTCATCATCGTCCTCAGTTC-3′ (see Figure 1A). The second round PCR was performed with the sense Universal Primer and two different antisense primers for amplification of different subset of *Slc4a10* transcripts, primer “b” 5′-gattacgccaagcttGCTCTTTCTTCCACCAAGCGGCAC-3′ (exon 6) for the 5′-UTR (common for all *Slc4a10* transcripts), primer “a” 5′-gattacgccaagcttGAAATGCTCACAGGTTCCAGGCTG-3′ (exon 4) specific for transcripts encoding MCDL-NBCn2 (lower case representing the artificially introduced sequence for cloning into pRACE vector).

Full-length cDNA encoding NBCn2 was amplified by nested PCR with cDNA of rat small intestine with primer set rNBCn2-F1 (5′-TGGTGAGTTGGAGTGTGCAGTTGCC-3′) and rNBCn2-R1 (5′-GGTGTTGACCTGCTCAGAGGCTGAAC-3′) for the 1st round PCR, and rNBCn2-F2 (5′-atcgcccgggCCTGATCCGAATACTAAGCAGAGCG-3′ and rNBCn2-R2 (5′-atcatgcggccgcACTTATGAAGGTGGATTTGGG ATGGG-3′) for 2nd round of PCR.

### Intracellular pH Measurement

The cDNA encoding rat NBCn2-K were subcloned into pGH19, an expression vector for *Xenopus* laevis oocytes. The expression vector for NBCn2-G was described previously (Liu et al., 2013). Both NBCn2-G and NBCn2-K were tagged with EGFP at the carboxyl termini. The plasmids were linearized and used for cRNA preparation with T7 mMessage mMachine^®^ kit (cat#AM1344, Life Technologies Corporation). 25 ng of cRNA was injected into oocyte of stages V-VI. The oocytes were incubated in OR3 medium for 4-5 days at 18 ^*o*^C before electrophysiology recordings.

Intracellular pH was measured with microelectrode as described previously (Musa-Aziz et al., 2010). Briefly, an oocyte was placed in a chamber and superfused with nominally HCO_3_^–^-free standard ND96 and then with 1.5% CO_2_/10 mM HCO_3_^–^. The oocyte was impaled with two microelectrodes: one proton-sensitive for monitoring of intracellular pH (pH_i_), and the other filled with 3 M KCl for monitoring membrane potential (*V*_m_) of the oocyte. The signals of pH_i_ and *V*_m_ electrodes were acquired by using a Hiz 223 (Warner Instruments, Hamden, CT, United States) dual-channel high-impedance electrometer (World Precision Instruments, Inc., Sarasota, FL, United States) and an OC-275 oocyte clamp (Warner Instrument Corp., Hamden, CT, United States). The pH_i_ of the oocyte is a linear function of the differential outputs of the two amplifiers.

### Solutions for Electrophysiology

#### OR3 Medium

One packet of L-15 medium was dissolved in 1.5 liter of H_2_O, followed by the addition of 100 ml of penicillin/streptomycin (10,000 Units/ml of penicillin, 10,000 μg/ml of streptomycin. Invitrogen, Carlsbad, California, United States) and 5 mM HEPES.

#### Standard ND96

Standard ND96: (in mM) 96 NaCl, 2 KCl, 1 MgCl_2_, 1.8 CaCl_2_, and 5 HEPES, pH 7.50.

#### Na-Free ND96

Na-free ND96: N-methyl-D-glucamine (titrated to pH 7.50 with HCl to generate NMDG^+^) replaced NaCl in standard ND96 solutions.

#### 1.5% CO_2_/10 mM HCO_3_^–^

1.5% CO_2_/10 mM HCO_3_^–^: (in mM) 86 NaCl, 2 KCl, 1 MgCl_2_, 1.8 CaCl_2_, and 5 HEPES; after adjusting pH to 7.50, we added 10 NaHCO_3_. The solution was bubbled with 1.5% CO_2_ balanced with O_2_.

### Western Blotting

A tissue of rat small intestine was placed in a glass tube containing pre-cooled protein isolation buffer (7.5 mM NaH_2_PO_4_, 250 mM sucrose, 5 mM EDTA, 5 mM EGTA, pH 7.0) containing 1% protease inhibitor cocktail (Cat#P8340; Sigma-Aldrich, St. Louis, MO, United States) and homogenized with a Glas-Col Teflon glass homogenizer (Glas-Col, Terre Haute, IN, United States). The crude homogenate was centrifuged for 10 min at 3,000*g*, 4°C. The supernatant was saved and centrifuged for 60 min at 100,000*g*, 4°C. The pelleted membrane fraction was dissolved with a buffer containing 20 mM Tris, 5 mM EDTA, 5% SDS, pH 8.0. The concentration of the membrane proteins was measured with Enhanced BCA Protein Assay Kit (Cat#P0010; Beyotime). The membrane proteins were stored in aliquots at −80°C until usage.

The membrane proteins were then separated by sodium dodecyl sulfate polyacrylamide gels (SDS-PAGE) and then blotted onto a PVDF membrane. The blot was blocked with 5% milk in 1 × TBST (1 mM Tris, 150 mM NaCl, 0.1%Tween 20, pH 7.4) for 1 h at room temperature (RT), and then probed with the primary antibody in 1 × TBST containing 1% milk at 4°C overnight. After five washes with 1 × TBST, the blot was incubated with HRP-conjugated secondary antibody in 1 × TBST containing 1% milk for 2 h at RT, followed by five washes with 1 × TBST. The blot was then incubated with SuperSignal West Pico chemiluminescent reagent (Thermo Scientific, Rockford, IL) for X-ray exposure.

### Immunofluorescence

Adult SD rats were anesthetized by subcutaneous injection of pentobarbital sodium and fixed by transcardial perfusion with 4% paraformaldehyde in PBS containing 77.4 mM Na_2_HPO_4_, 22.6 mM NaH_2_PO_4_, pH 7.4. Small intestine was collected and stored in PBS supplemented with 0.1% PFA and 0.02% NaN_3_. A cryo-section of 5 μm was baked at 60°C overnight, rehydrated in 1 × TBS (1 mM Tris, 150 mM NaCl, pH 7.4) at RT for 1 hr, followed by 5 washes with 1 × TBS. The section was then incubated at 98°C for 20 min in Improved Citrate Antigen Retrieval Solution (catalog no. P0083; Beyotime). After five washes with 1 × TBS, the section was blocked for 2 h with 5% normal goat serum in 1 × TBS, and then incubated with primary antibody at 4°C overnight. The section was washed 5 min × 5 times with 1 × TBS and incubated with DyLight-conjugated secondary antibody at RT for 1 h. After three washes with 1 × TBS, the section was counterstained with 4′,6-Diamidino-2-Phenylindole (DAPI) at room temperature for 5 min, washed three times with 1 × TBS, and mounted with Antifade Polyvinyl Pyrrolidone Medium (cat#P0123; Beyotime). Images were acquired on a FluoView FV1000 confocal microscope (Olympus, Tokyo, Japan).

### Statistics

Quantitative data are represented as mean ± SEM. Student’s *t*-test was performed with Microsoft Excel. One-way ANOVA analysis was performed with Minitab (Minitab Inc.).

## Results

### 5′-RACE and Cloning of Full-Length NBCn2 From Rat Intestine

In the previous study, NBCn2 protein was detected from the small intestine of rat by using an antibody specific to MCDL-NBCn2 (Liu et al., 2013). Intriguingly, we were not able to amplify the cDNA encoding MCDL-NBCn2 from the small intestine of rat by using primers specific to the known MCDL-NBCn2 variants (data not shown). It is likely that NBCn2 in rat small intestine was expressed by a mechanism different from the known MCDL-NBCn2. Thus, we performed 5′-RACE with the total RNA preparation from the small intestine of rat.

**FIGURE 2:**
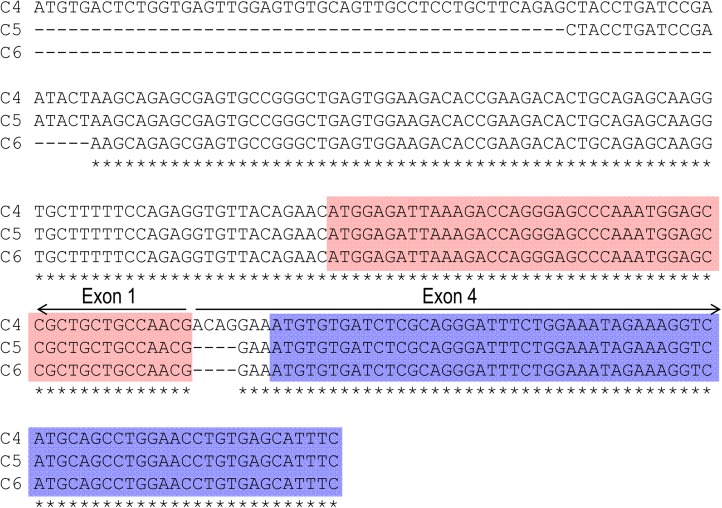
Sequence alignment of three partial clones obtained by 5′-RACE. All three clones of C4, C5, and C6 include exon 1 (encoding “MEIK”) and exon 4 (encoding “MCDL”). Clone C4 contains additional 4 nucleotides (nt) “ACAG” at the 5′-end of exon 4, and thus is predicted to express MCDL-NBCn2. Both clones 5 and 6 contain exon 1 and exon 4 in frame, and thus are predicted to express MEIK-NBCn2. Stars indicate the sequences identical in all three clones. The red boxes indicate the coding region of exon 1 for “MEIK⋅⋅⋅⋅⋅⋅”, whereas the blue boxes indicate the coding region of exon 4 for “MCDL⋅⋅⋅⋅⋅⋅”.

**FIGURE 3:**
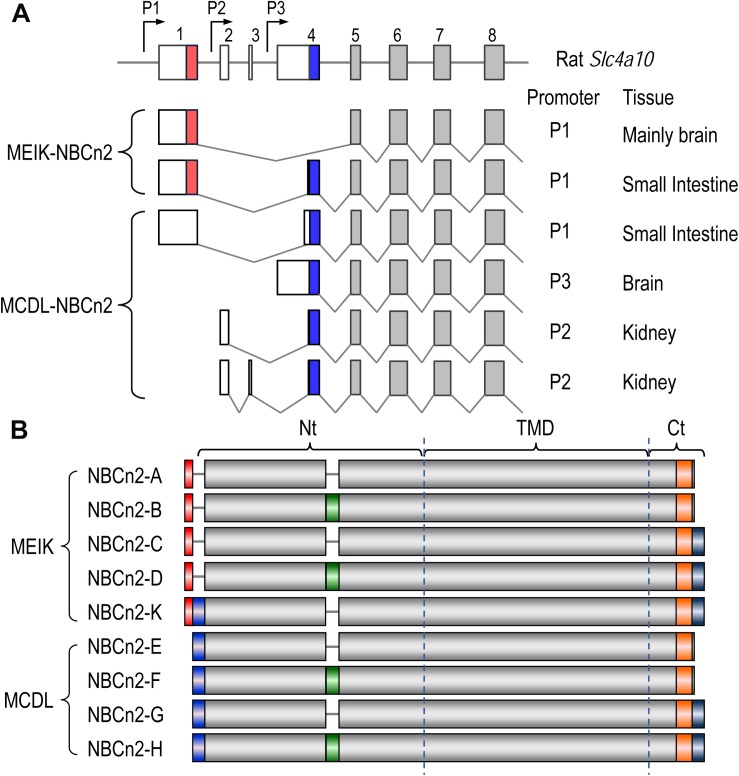
Diagrams of partial-exon structures of rat *Slc4a10* transcripts and full-length variants of NBCn2. **(A)** Partial-exon structures of *Slc4a10* transcripts. **(B)** Diagrams of predicted primary structures of full-length NBCn2 variants. Not shown here are two full-length variants identified in mouse NBCn2-I and -J with minor variation at the Ct end, as well as four Nt-truncated NBCn2 variants originally identified from rat kidney (Wang et al., 2015). NBCn2-K is predicted to start with “MEIK”. However, the NBCn2 in small intestine can be detected with anti-MCDL, but not with anti-MEIK (see text in “Discussion”).

Figure 1A represents the diagram of the structure of rat *Slc4a10*. As shown in Figure 1B, by 5′-RACE using a primer complimentary to a sequence in exon 6 of rat *Slc4a10*, we obtained a band of ∼400 bp. From this 400-bp product, we identified two clones that are predicted to encode a regular NBCn2 starting with “MEIK”. By 5′-RACE using a primer complimentary to a sequence in exon 4 of rat *Slc4a10*, we obtained a product of ∼350 bp. From this 350-bp product, we identified three clones, designated as C4, C5, and C6. All three clones contain exon 1 (containing the coding sequence for “MEIK”) and exon 4 (containing the coding sequence for “MCDL”). C5 and C6 are predicted to express an NBCn2 starting with “MEIK” but also containing “MCDL”. C4 contains “ACAG” at the 5′-end of exon 4 due to alternative splicing using a cryptic splicing donor site of exon 4, thus is predicted to encode an NBCn2 protein starting with “MCDL”. Figure 2 shows the sequence alignment of the three partial clones C4, C5, and C6 of *Slc4a10* obtained by 5′-RACE. Figure 3A summarizes the partial exon structures of *Slc4a10* transcripts encoding the known full-length NBCn2 variants.

Based upon the 5′-RACE results, we performed cDNA cloning for NBCn2 from rat small intestine by nested RT-PCR using a set of primers specific to MEIK-NBCn2. Figure 1C shows a representative result of the nested RT-PCR. From the band of ∼3.5 kb, we identified a full-length cDNA encoding the known NBCn2-C (accession #AAO59639) as well as a novel full-length cDNA encoding NBCn2-K (Accession #KY703228). This cDNA of NBCn2-K contains a 5′-UTR similar to the above-mentioned clones C5 and C6, thus it is predicted to express an NBCn2 starting with “MEIK”, while containing “MCDL”.

Figure 3B summarizes the diagram of primary peptide structures of known full-length NBCn2 variants. In summary, *Slc4a10* can express two groups of full-length NBCn2 variants, one starting with “MEIK” and the other “MCDL”. NBCn2-K represents the novel full-length NBCn2 variant identified from rat small intestine in the present study.

### Functional Characterization of NBCn2 Variants in Xenopus Oocytes

Rat NBCn2 was heterologously expressed in *Xenopus* laevis oocytes. An oocyte was placed in a perfusion chamber. The oocyte was first superfused with norminally “HCO_3_^–^ free” ND96 solution and then with a solution containing 5% CO_2_/33 mM HCO_3_^–^. The intracellular pH (pH_i_) and membrane potential (*Vm*) of the oocyte were monitored simultaneously with two microelectrodes impaled into the cell.

**FIGURE 4:**
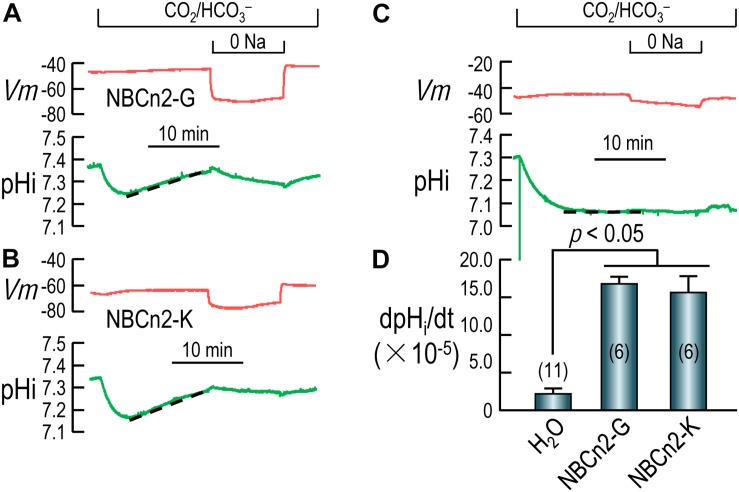
Functional characterization of NBCn2 in *Xenopus* oocytes. **(A)** Representative recordings of *Vm* and pH_i_ of an oocyte expressing rat NBCn2-G. **(B)** Representative recordings of *Vm* and pH_i_ of an oocyte expressing rat NBCn2-K. **(C)** Representative recordings of *Vm* and pH_i_ of a control oocyte injected with H_2_O. **(D)** Summary of pH_i_ recovery rate dpH_i_/dt. The dpHi/dt of both NBCn2-G and NBCn2-K are not significantly different from each other, but both are significantly higher than that of H_2_O-injected control oocytes by one-way ANOVA analysis followed by Fisher’s comparison. The numerals in the parentheses in panel **D** indicate the number of individual oocytes included in each bar.

Figures 4A,B show the typical recordings of pH_i_ and *Vm* of oocytes expressing NBCn2-G and NBCn2-K, respectively. Upon the introduction of CO_2_/HCO_3_^–^, the pH_i_ of the cell undergoes a rapid fall due to the influx of CO_2_, and then a modest recovery due to the influx of HCO_3_^–^ via NBCn2, contrasting to the lack of pHi recovery in H_2_O-injected control cell (Figure 4C). The rate of pH_i_ recovery (dpH_i_/dt) is an index of the HCO_3_^–^-transport activity of NBCn2. As summarized in Figure 4D, the dpH_i_/dt of NBCn2-K is not significantly different from that of NBCn2-G. The dpH_i_/dt of both NBCn2-G and -K are significantly higher than that of the control cells injected with H_2_O.

### Tissue and Cellular Expression of NBCn2 in Rat Small Intestine

**FIGURE 5:**
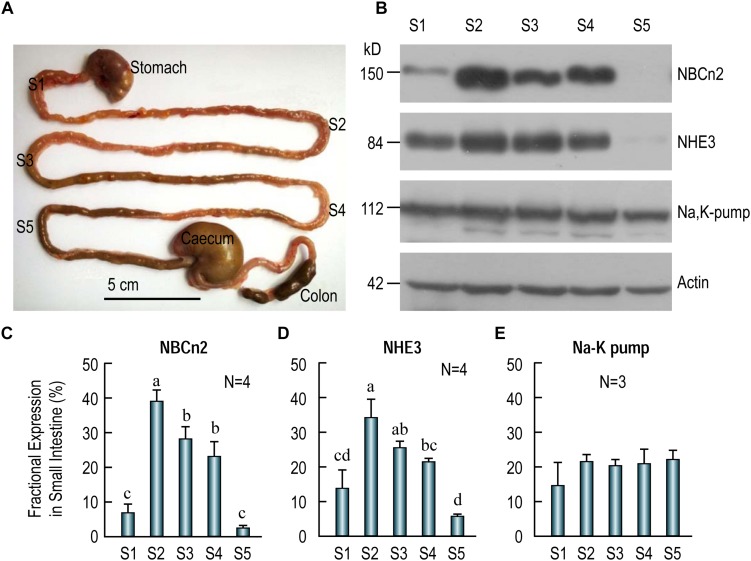
Distribution of NBCn2, NHE3, and Na^+^-K^+^ pump in different segments of rat small intestine. **(A)** Image showing the tissue collection sites for different fragments of the small intestine. S1, duodenum; S2, proximal jejunum; S3, middle jejunum; S4, distal jejunum; S5, ileum. Scale bar, 5 cm. **(B)** Western blotting of NBCn2 (with anti-MCDL), NHE3, and Na^+^-K^+^ pump in segments S1-S5 of the small intestine. β-actin is used as loading control. **(C)** Fractional distribution of NBCn2 in S1-S5. **(D)** Fractional distribution of NHE3 in S1-S5. **(E)** Fractional distribution of Na^+^-K^+^ pump in S1-S5. The density of NBCn2 was normalized to that of actin of the same lane from blots like those shown in panel **(B)**. The fractional distribution of NBCn2 in each segment was computed by dividing this normalized density by the sum of the normalized densities of S1-S5. The fractional distribution of NHE3 and Na-K pump was computed by a similar approach. Ns in panels **C–E** indicate the number of rat individuals included in each panel. One-way ANOVA followed by Fisher’s comparison was used for statistical analysis. *P* < 0.05 is considered significantly different. Bars not marked by a same alphabet are significantly different from each other.

The small intestine is commonly divided into three different segments, namely, duodenum, jejunum, and ileum. In rat, the jejunum accounts for about 90% length of the small intestine (Kararli, 1995; Sharp and Villano, 2012). To examine the distribution of transporters along the small intestine, we sampled tissues from five different segments, S1-S5, in the small intestine of adult rat (see diagram in Figure 5A). S1 represents duodenum. S2, S3, and S4 represent the proximal, middle and distal jejunum, respectively. Finally, S5 represents ileum.

Figure 5B shows the representative results of western blotting for NBCn2 (full-blot shown in Supplementary Figure S1), NHE3, and Na^+^-K^+^-pump. As summarized in Figure 5C, the fractional expression of NBCn2 is highest in the jejunum (S2-S4) is enriched in the jejunum from segments S2-S4, and to a much lesser extent in S1 (i.e., duodenum), but not detectable in S5 (i.e., ileum). Similarly, the fractional expression of NHE3 is highest in jejunum (S2-S4), lower in duodenum, and lowest in ileum (Figure 5D). In contrast, the Na^+^-K^+^ pump is virtually equally expressed in duodenum, jejunum, and ileum along the entire small intestine (Figure 5E).

Our data indicate that NBCn2, along with NHE3, is mainly expressed in the jejunum, and are rarely detectable in ileum in the small intestine of adult rats. It is interesting that a previous study by Northern blotting showed the expression of NBCn2 mRNA in rat ileum (Wang et al., 2000). It was not clear exactly which portion of rat small intestine the authors used for the northern blotting study. Note that, our western blotting data show that NBCn2 is expressed in about 3/4 along the length of the small intestine in rat.

**FIGURE 6:**
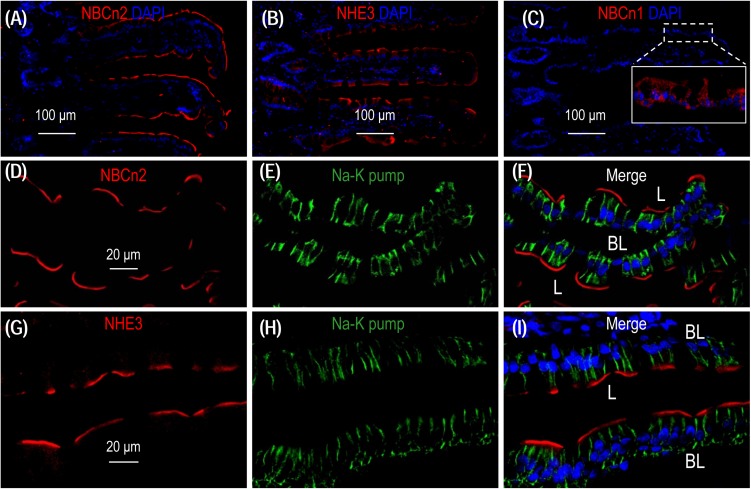
NBCn2 and NHE3 are expressed at the apical membrane of epithelium in small intestine. **(A)** Overview of staining with anti-MCDL (NBCn2) in the small intestine. **(B)** Overview of staining with anti-NHE3 in the small intestine. **(C)** Negative staining of anti-NBCn1 in the small intestine. In these experiments, the final concentrations of anti-MCDL, anti-NHE3, and anti-NBCn1 for immunofluorescence staining were 1.5 μg/ml (1:400 dilution). NBCn2 and NHE3 are mainly expressed in the villi of the small intestine. In panel **C**, no significant staining was observed for anti-NBCn1 when visualized under microscopy with parameters the same as those used for panel **A** and **C**. Inset in panel **C** shows that the non-specific background staining by anti-NBCn1, when visualized by a much higher exposure, is distributed throughout the cytosol of the epithelia. **(D–F)** High magnification view shows that NBCn2 is exclusively expressed at the apical membrane of small intestine epithelium. **(G–I)** High magnification view shows that NHE3 is exclusively expressed at the apical membrane of small intestine epithelium. In these experiments, the basolateral membrane is stained by α1 of Na^+^-K^+^ pump.

To examine the localization of NBCn2 and NHE3 in the small intestine of rat, we performed immunofluorescence with cryo sections of rat small intestine. An overview shows that the signal derived from anti-NBCn2 is mainly localized in the villi of the small intestine (Figure 6A). The same is true for anti-NHE3 (Figure 6B). In a control experiment, we stained a cryo-section of the small intestine with rabbit polyclonal anti-NBCn1. When visualized by using the same exposure parameters on the confocal microscopy, no signal was observed in the epithelia of the small intestine (Figure 6C). Inset in Figure 6C shows the non-specific background staining by anti-NBCn1 throughout the cytosol visualized by a much stronger exposure. The data indicate that the fluorescence signals derived from anti-NBCn2 and anti-NHE3 are specific for NBCn2 and NHE3, respectively.

A high magnification view shows that, when co-stained with anti-α1 against the α1 subunit of Na^+^-K^+^ pump, a specific marker for the basolateral membrane of the epithelium, NBCn2 is exclusively expressed in the apical domain of the absorptive columnar enterocytes (Figures 6D-F). The same is true for NHE3 (Figures 6G-I). Our data for the cellular distribution of NHE3 in the small intestine is consistent with previous studies (Murer et al., 1976; Hoogerwerf et al., 1996).

High salt intake decreases expression of NBCn2 and NHE3 in rat small intestine.

We examined the effect of high salt diet on the expression of NBCn2 in the small intestine. The rats were fed with normal chow and water containing 1.5% NaCl for 7 days. As summarized in Table 1, the average daily H_2_O consumption of the NaCl group is significantly higher than that of the control group. The average food intake and the body weights before (day 0) and after (day 7) treatment are not significantly different between the NaCl group and the control.

**TABLE 1:** Average H_2_O and food consumption, and body weight of the NaCl and control groups.

	**N**	**H_2_O (ml/d)**	**Food (g/d)**	**Weight (g)/day0**	**Weight (g)/day7**
NaCl	8	45.1 ± 2.2	20.5 ± 0.5	186.0 ± 1.3	207.3 ± 3.8
Control	8	31.1 ± 1.9	20.5 ± 0.6	185.6 ± 1.4	209.6 ± 2.9
*p*-value	/	<0.001	NS	NS	NS

*NS, not significant; Two-tailed unpaired student’s *t*-test was performed for statistical analysis.*

**FIGURE 7:**
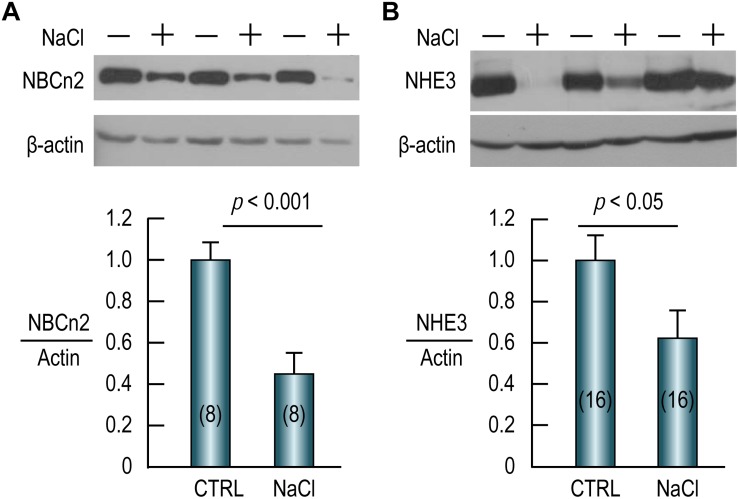
High NaCl intake decreases expression of NBCn2 **(A)** and NHE3 **(B)** in small intestine of rat. Membrane preparations of the small intestine were used for western blotting to examine the expression of NBCn2 (probed with anti-MCDL) or NHE3. The density of the target transporter in each lane was normalized to that of actin of the same lane. This ratio of each lane was then normalized to the average of the ratios of the control lanes in the same blot. Compared to the control, NaCl treatment decreases the abundance of NBCn2 by 55% and that of NHE3 by 40%. Numerals in the parenthesis indicate the numbers of rats included in each condition. For statistical comparison, two-tailed student’s *t*-test was performed. “−” indicates the controls, whereas “+” indicates the rats treated with NaCl.

As shown in Figure 7A (full-blot shown in Supplementary Figure S2), compared to the control, the abundance of NBCn2 in the small intestine is reduced by 55% by NaCl treatment. Similarly, the abundance of NHE3 in the small intestine is reduced by ∼40% in the NaCl group compared to the control (Figure 7B). Our data indicate that NBCn2 and NHE3 likely play an important role in the transport of NaCl in the intestinal epithelium.

## Discussion

The rat *Slc4a10* gene contains three promoters (Wang et al., 2015), see Figure 1A). Promoter P1 of *Slc4a10* is most active in the brain, but is also expressed in many other tissues, such as the eye and the kidney. The transcripts derived from P1 could be translated into two different groups of NBCn2 variants: one starting with “MEIK” (mostly lacking “MCDL”, exception the novel NBCn2-K) and the other starting with “MCDL”. Promoter P2 is active in the kidney, producing NBCn2 variants starting with “MCDL” (Liu et al., 2013; Wang et al., 2015). Finally, promoter P3 is active in the brain, producing NBCn2 variants starting with “MCDL” (Wang et al., 2015).

In the present study, the novel *Slc4a10* transcript encoding NBCn2-K contains in-frame both exon 1 (encoding “MEIK”) and exon 4 (encoding “MCDL”). However, by western blotting with protein preparations of the small intestine of rat, NBCn2 protein could be detected only with anti-MCDL (Figure 5), but not with anti-MEIK (data not shown). It is possible that the *in vivo* translation of NBCn2 proteins in the small intestine is initiated at “MCDL” rather than at “MEIK”.

In rat, similar to NHE3, the expression of NBCn2 is enriched in the apical domain of jejunum along the small intestine. The apical localization of NBCn2 is of great interest. In the following paragraphs, we will address the potential physiological role of NBCn2 in the NaCl absorption by small intestine epithelium.

Nutrients and water must across the barrier of the gastrointestinal epithelia layer to enter the body. The small intestine is the major absorber of electrolyte (e.g., Na^+^ and Cl^–^) and fluid along the entire gastrointestinal tract. Fluid absorption is largely driven by the absorption of Na^+^, HCO_3_^–^, and Cl^–^. A series of functional studies have indicated that the absorption of Na + and HCO_3_^–^ is closely associated in the small intestinal epithelium. On one hand, HCO_3_^–^ greatly stimulates the absorption of Na^+^ in the small intestine of both human (Fordtran et al., 1968; Turnberg et al., 1970a, b) and rat (Esposito and Csaky, 1977; Humphreys and Chou, 1983; Patra et al., 1989). On the other hand, Na^+^ also stimulates the absorption of HCO_3_^–^ in rat jejunum (Hubel, 1973).

**FIGURE 8:**
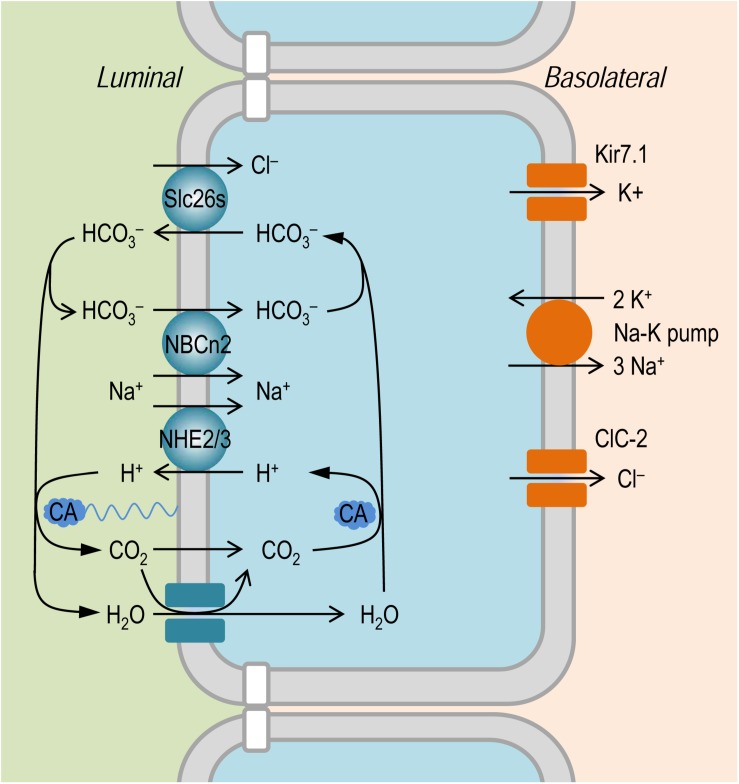
Model to show the hypothetical role of NBCn2 in small intestine epithelium. In the apical membrane, NHEs (e.g., NHE2 and NHE3) in concert with SLC26 transporters mediate NaCl absorption. The NaCl absorption via this pathway involves the action of carbonic anhydrase (CA) in both the luminal side and the cytosol. The presence of NBCn2 in the apical membrane likely provides an alternate pathway for the absorption of Na^+^ and the direct uptake of luminal HCO_3_^–^. In the basolateral membrane, NaCl extrusion is mediated by the coupled action of Na^+^-K^+^ pump and Cl^–^ channel ClC-2 (Lipecka et al., 2002; Pena-Munzenmayer et al., 2005). Kir7.1 expressed in the basolateral membrane provides a pathway for the cycling of K^+^ (Partiseti et al., 1998; Nakamura et al., 1999).

It is established that the NaCl absorption in the intestinal epithelium involves the coordinated action of the apical Na^+^/H^+^ exchangers, such as NHE2 and NHE3, of Slc9 family (Murer et al., 1976; Collins et al., 1993; Hoogerwerf et al., 1996; Wormmeester et al., 1998; Xia et al., 2014), and the anion exchangers, such as Slc26a3 and Slc26a6, of Slc26 family (Wang et al., 2002; Gawenis et al., 2004; Xia et al., 2014); for review, see Kato and Romero (2011). As shown in Figure 8, the Slc26 anion exchangers mediate the absorption of Cl^–^ in exchange of intracellular HCO_3_^–^. The apical NHEs mediate the entry of Na^+^ in exchange of intracellular H^+^. The HCO_3_^–^ secreted by the Slc26 anion exchangers is titrated by the proton secreted via NHEs to form CO_2_ under the influence of membrane associated carbonic anhydrase (CA). The CO_2_ would then enter – either by simple diffusion or via membrane channels such as aquaporins (Laforenza et al., 2009; Suzuki, 2010) that are permeable to gas molecules as tested in model cells (Endeward et al., 2006; Musa-Aziz et al., 2009; Geyer et al., 2013) – the epithelium to reconstitute H^+^ (for recycling by the NHEs) and HCO_3_^–^ (for recycling by the Slc26s) under the influence of cytosol CA. During the absorption of NaCl, the influx of Na^+^ via apical NHEs is driven by the inward electrochemical gradient established by the basolateral Na^+^-K^+^ pump. In the small intestinal epithelium, NHE3 encoded *SLC9A3* presumably makes a major contribution to the absorption of NaCl. Indeed, mutations of *SLC9A3* in human (Janecke et al., 2015) and genetic disruption of *Slc9a3* in mouse (Schultheis et al., 1998; Gawenis et al., 2002; Woo et al., 2003) cause diarrhea.

Note that, at least in human jejunum, CA inhibitor acetazolamide (at high concentration of 500 mg/L = 2,250 μM) decreases just by 50% the rate of HCO_3_^–^ absorption (Turnberg et al., 1970b). This observation indicates that the HCO_3_^–^ absorption in the human jejunum contains a substantial component that is independent of the pathway mediated by the NHEs + CA.

The apical NBCn2 could provide an alternate pathway for direct uptake of Na^+^ and HCO_3_^–^ into the small intestinal epithelium. In principle, driven by the inward Na^+^ electrochemical gradient under physiological condition, the electroneutral NBCn2, just like the electroneutral NHE3, would certainly mediate influx of Na^+^. As an acid extruder, NBCn2 is somewhat equivalent to NHE3 + CA, although the two systems (NBCn2 vs. NHE3 + CA) each might have different efficacy in mediating the transmembrane fluxes of acid-base equivalents (Guo et al., 2017).

We hypothesize that NBCn2, coupled with the Slc26 anion exchangers, mediate NaCl absorption in the small intestine. As shown in Figure 8, here, NBCn2 mediates the direct uptake of ions Na^+^ and HCO_3_^–^. The HCO_3_^–^ is then extruded by the Slc26 anion exchangers for the uptake of the luminal Cl^–^. Thus, the apical NBCn2, coupled with Slc26s, provides an alternate pathway for NaCl absorption that involves no CA. In the present study, the down regulation of NBCn2 induced by high NaCl intake would reduce the capacity of the small intestine for the absorption of NaCl, which in turn would reduce the systemic NaCl input in the body, an adaptive response to the increased NaCl load into the gastrointestinal tract.

Functional studies with both human (Fordtran et al., 1968) and rat (Humphreys and Chou, 1983) show that HCO_3_^–^ stimulates the absorption of Na^+^ in the jejunum, but not in the ileum. In the present study, the restricted expression of NBCn2 and NHE3 in the jejunum, but not in the ileum, appears to be consistent with these previous functional studies.

The hypothetical role of NBCn2 in NaCl absorption could provide a teleological explanation for why NaCl overload down-regulates the expression of intestinal NBCn2, presumably in the apical domain of the small intestine. Decreasing the abundance of apical NBCn2 would reduce the capacity of the small intestine for NaCl absorption, therefore reducing the entry of NaCl and fluid volume into the body. In human, genetic study has shown that *SLC4A10* is associated with dysregulation of plasma osmolality and systemic water balance (Boger et al., 2017). It is possible that the pathological development of this phenotype in human contains an intestinal component involving NBCn2. It is true that no deficit in NaCl absorption has been reported for NBCn2-KO mice. It remains to be addressed whether NBCn2 is expressed in the small intestine of mice.

Finally, the mechanism underlying the regulation of the expression of intestinal NBCn2 and NHE3 by high salt diet remains to be addressed. The NaCl absorption in the small intestine is regulated by hormones (for review, see Kato and Romero, 2011). For example, glucocorticoids enhance (Charney et al., 1975), whereas atrial natriuretic peptide inhibits (Matsushita et al., 1991; Gonzalez Bosc et al., 2000) the absorption of NaCl and water in the small intestine. Glucocorticoids stimulate the expression and activity of NHE3 in the small intestine (Kandasamy and Orlowski, 1996; Yun et al., 2002). It is intriguing whether the decrease in intestinal NBCn2 and NHE3 in response to high salt diet involves the action of hormones.

In summary, we identify a novel NBCn2 variant in the small intestine of rat. This NBCn2 is expressed in the apical domain of the small intestinal epithelium. The abundance of NBCn2 is decreased by high NaCl intake. We hypothesize that NBCn2 represent a novel pathway for NaCl absorption in the small intestine. Further studies are necessary to address the physiological significance of NBCn2 in NaCl absorption in the epithelium of the small intestine.

## Data Availability Statement

The datasets generated for this study can be found in the GenBank, accession #KY703228.

## Ethics Statement

The animal study was reviewed and approved by the Institutional Committee on Animal Care and Use at Huazhong University of Science and Technology.

## Author Contributions

J-LW and M-JR performed the molecular cloning. J-LW and JZ performed the western blotting and immunofluorescence. MW contributed to the animal models. D-KW performed the pH_i_ measurement. YL, LZ, WB, and L-MC designed the study. YL and L-MC wrote the manuscript. All authors approved the manuscript.

## Conflict of Interest

The authors declare that the research was conducted in the absence of any commercial or financial relationships that could be construed as a potential conflict of interest.
