# Supplementary material for: Expression, Localization, and Effect of High Salt Intake on Electroneutral Na+/HCO3– Cotransporter NBCn2 in Rat Small Intestine: Implication in Intestinal NaCl Absorption
Source: Front Physiol. 2019 Oct 29;10:1334. doi: 10.3389/fphys.2019.01334 (PMC6828735; doi:10.3389/fphys.2019.01334)
Supplement: Supplementary file 1 [file Data_Sheet_1.PDF]

Supplementary FIGURE S1

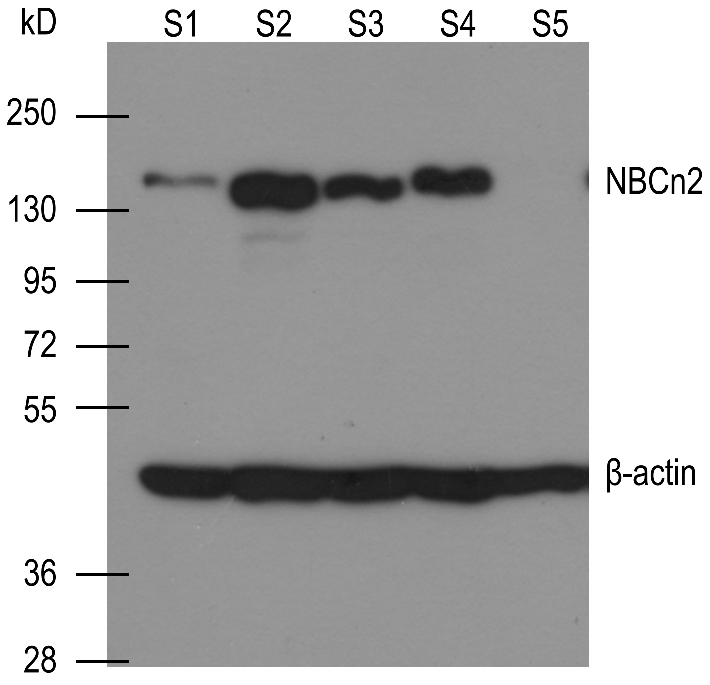

The full-blot for the NBCn2 in Figure 5B. The blot was simultaneously probed with anti-MCDL and anti-actin. Anti-MCDL virtually recognizes a single band with a molecular weight of about 150 kD.

Supplementary FIGURE S2

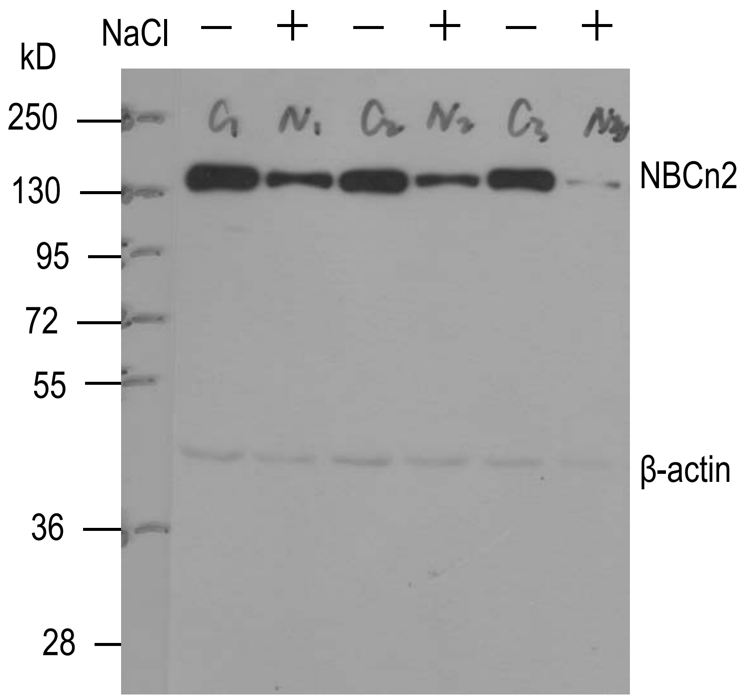

The full-blot for the NBCn2 in Figure 7A. The blot was simultaneously probed with anti-MCDL and anti-actin. Anti-MCDL virtually recognizes a single band with a molecular weight of about 150 kD.
